# A Comparative Analysis of AI HRM Governance Approaches Across African Countries: Continental Patterns and Divergences

**DOI:** 10.12688/f1000research.184277.2

**Published:** 2026-08-31

**Authors:** Samuel Bangura, Melanie Elisabeth Lourens

**Affiliations:** 1 Department of Human Resource Management, Faculty of Management Science, Mangosuthu University of Technology, Umlazi, KwaZulu-Natal, 4031, South Africa; 2 Faculty of Management Science, Durban University of Technology, Durban, KwaZulu-Natal, 4001, South Africa

**Keywords:** AI HRM governance, Africa, comparative institutionalism, data protection, employment regulation, Ubuntu, African Union, digital transformation, polycentric governance, AI ethics

## Abstract

**Background:**

The governance of artificial intelligence within human resource management across the 54 sovereign states of Africa presents distinctive challenges that differ markedly from those observed in Euro-American contexts. African organizations have adopted AI-driven workforce tools at annual growth rates exceeding 25% since 2018; however, governance frameworks remain in their nascent stages and are characterized by fragmentation. Current comparative scholarship has largely overlooked innovative governance developments within the African continent, often positioning Africa as a recipient rather than an active innovator in AI regulation.

**Methods:**

This study employed a qualitative comparative analysis encompassing eleven focal countries, representing five regional clusters. Data sources included legislative documents, professional standards, civil society instruments, and expert practitioner insights. The analysis focused on five key governance dimensions: legislative architecture, professional standards, digital infrastructure, philosophical traditions, and mechanisms established by civil society.

**Results:**

Three overarching governance patterns emerged across the continent: (1) the functioning of data protection laws as de facto governance frameworks for AI in human resource management; (2) the significant influence exerted by international development finance institutions on national AI policy formulation; and (3) ongoing tensions between digital transformation aspirations and institutional capacity limitations. Variations across countries were structured along three axes: the specificity of regulations pertaining to AI-driven employment decisions; the integration of African philosophical traditions such as Ubuntu, Harambee, Ma’at, and Teranga; and the degree of regional economic community harmonisation initiatives. Based on these insights, the African AI HRM Governance Typology classified countries into four ideal types:

**Conclusions:**

Achieving effective AI HRM governance across Africa necessitates tailored harmonisation strategies that respect the varying levels of governance maturity. Critical actions include the development of an African Union Model Law on AI in Employment, the establishment of minimum standards within the framework of the African Continental Free Trade Area, and the promotion of regional economic community governance coalitions. Future research should focus on empirically assessing governance effectiveness, exploring employer practices across the continent, and analysing the impact of trade liberalisation on regulatory convergence.

## Introduction

The governance of artificial intelligence in human resource management sits at the intersection of two profound global challenges: the need to harness the productive potential of AI-driven workforce technologies, and the imperative to prevent those technologies from perpetuating or amplifying systemic inequality, discriminatory employment practices, and erosions of worker dignity. Across the African continent, these challenges are mediated by governance contexts that differ substantially from the Euro-American institutional environments in which most AI HRM scholarship and regulation have been generated. Africa’s 54 sovereign states collectively encompass an extraordinary diversity of legal traditions, institutional capacities, digital infrastructure levels, labour market structures, and cultural and philosophical orientations. This diversity makes any single governance prescription ill-suited to the continent, and that demands comparative analytical frameworks capable of capturing both cross-national patterns and significant divergences. The urgency of this analysis is underscored by the pace of AI workforce technology adoption across African organisations. Existing comparative scholarship on AI governance has focused primarily on contrasts between the European Union’s regulatory approach, the United States’ sector-specific model, China’s state-led developmentalism, and, to a lesser extent, the governance dynamics of major emerging economies, including India and Brazil ( Cihon et al., 2020; Roberts et al., 2021). The article makes four principal contributions. First, it provides the most comprehensive comparative mapping to date of AI HRM governance frameworks across five African regional clusters and eleven focal countries. Second, it identifies three continental governance patterns and three axes of divergence that structure the comparative landscape. Third, it introduces the African AI HRM Governance Typology (AAHGT), an original classificatory framework for characterising country-level governance approaches. Fourth, it develops implications for continental governance harmonisation through the African Union’s institutional architecture, with reference to the AfCFTA and the African Union Digital Transformation Strategy for Africa. The article is structured as follows. Section 2 establishes the theoretical framework. Section 3 presents the comparative methodology. Sections 4 through 8 examine governance approaches across the five regional clusters. Section 9 synthesises continental patterns and divergences. Section 10 introduces the AAHGT. Section 11 develops implications for harmonisation. Section 12 concludes.

## Theoretical Framework

### Comparative Institutionalism

Comparative Institutionalism, as elaborated by Hall and Soskice (2001) in their Varieties of Capitalism framework and extended to developing economies by Hancke et al. (2007) and Wood and Frynas (2006), provides a foundational theoretical lens for this analysis. The framework holds that the governance of economic activities, including workforce management, is shaped by the institutional complementarities that characterise national political economies: the interactions among legal systems, financial structures, industrial relations frameworks, educational and training systems, and inter-firm coordination mechanisms. Different national institutional configurations generate different incentive structures for employers, workers, and technology providers, producing systematic cross-national variation in governance outcomes even when the underlying technologies are identical.

The application of Comparative Institutionalism to African AI governance requires careful adaptation. Hall and Soskice’s original framework was developed from OECD country comparisons and privileges formal institutional configurations. African political economies are characterised by significant institutional hybridity, which is the coexistence of formal statutory institutions with customary governance traditions, informal economic norms, and externally imposed regulatory frameworks inherited from colonial administration ( Beyer, 2005). AI HRM governance in African contexts emerges from the interplay of these heterogeneous institutional layers in ways that comparative institutionalism, as traditionally formulated, does not fully anticipate.

### Institutional Hybridity Framework

The Institutional Hybridity Framework provides a comprehensive perspective for understanding the coexistence and interaction of customary, colonial, and statutory legal systems in shaping AI-enabled human resource management (HRM) governance within African contexts, each exerting distinct influences on organizational practices. Customary law, grounded in traditional norms and community-centered values, continues to influence local HRM processes such as recruitment and conflict resolution. Conversely, colonial law, characterized by historically hierarchical and exclusionary structures, has engendered enduring biases impacting gender relations and labor segmentation ( Fechner, 2022). Simultaneously, statutory law embodies modern regulatory efforts to synthesize these diverse legal traditions, fostering formal employment standards and compliance in HRM. As artificial intelligence technologies become increasingly integrated into HR functions, they present pressing ethical challenges, notably the potential reinforcement of colonial-era biases unless effectively mitigated through alignment with local knowledge systems and principles of fairness ( Makhanya, 2025). Effective governance in this domain necessitates robust stakeholder engagement, involving HR professionals, technologists, and other relevant actors to collaboratively manage associated risks ( Ramadianto et al., 2025). Additionally, decolonial perspectives emphasize the importance of incorporating epistemic justice to ensure that technological implementations respect and embody local values and realities ( Makhanya, 2025). Ultimately, while the Institutional Hybridity Framework elucidates promising opportunities for innovative governance via legal pluralism, it also highlights the ongoing challenge of reconciling historical injustices with rapid technological advancements, underscoring the necessity for continuous ethical vigilance and inclusive, participatory governance in this evolving landscape.

### Polycentric Governance Theory

Polycentric Governance Theory, originating in Ostrom’s (2010) work on common-pool resource management and extended to AI governance by Marchetti (2021) and Dafoe (2018), complements comparative institutionalism by foregrounding the multiplicity of governance actors and levels relevant to AI regulation. Rather than treating governance as a state-centric, top-down regulatory enterprise, polycentric theory recognises that effective AI governance in practice involves the coordinated contributions of actors at multiple levels: international organisations (African Union, United Nations, OECD), regional economic communities (ECOWAS, SADC, EAC, COMESA), national governments and regulatory bodies, professional associations, civil society organisations, technology vendors, and affected communities including workers and job-seekers. In African contexts, traditional governance institutions and community-based organisations add further layers to this polycentric architecture.

Polycentric theory generates a key analytical insight for this study: the effectiveness of AI HRM governance is not determined by the existence of any single legislative instrument but by the coherence and complementarity of governance contributions across levels and actors. A country may lack dedicated AI employment regulation but possess a robust data protection framework, a strong professional HR standards body, and active civil society engagement with AI ethics. Therefore, a polycentric governance configuration may outperform a country with a comprehensive AI law, but weak institutional capacity for enforcement and stakeholder engagement.

### African Philosophy as Governance Epistemology

A third theoretical strand grounds the analysis in African philosophical traditions as sources of governance epistemology, which can act as frameworks for defining what legitimate AI governance means in African contexts. The Ubuntu philosophy provides one such framework, foregrounding relational accountability, communal deliberation, and human dignity as governance values. Alongside Ubuntu, the analysis draws on Ujamaa (Tanzanian communitarian philosophy emphasising solidarity and collective ownership), Harambee (Kenyan cooperative self-help philosophy), Egyptianist Ma’at (the ancient Egyptian ethical concept of truth, balance, and social harmony, which continues to inform Egyptian ethical discourse), and the West African concept of Sankofa (the wisdom of learning from the past to navigate the present). The concepts represent selected indigenous governance epistemologies across various regions. They are regarded as philosophical traditions that transcend superficial cultural references, constituting authentic governance epistemologies that shape the legitimisation, deliberation, and contestation of artificial intelligence regulation within diverse African contexts. Consequently, they are deemed to hold significant importance. It is also noteworthy that these concepts are invoked by state actors rather than solely by non-governmental organisations.

## Methods

This article employs a qualitative comparative analysis (QCA) approach, informed by QCA logic drawing on the Most Similar Systems Design (MSSD) and Most Different Systems Design (MDSD) traditions in comparative political science ( Przeworski & Teune, 1970). The MSSD is applied within regional clusters, comparing countries that share regional economic community membership, colonial heritage, and broad development characteristics, to isolate the variables that account for within-cluster governance variation. The MDSD is applied across regional clusters to identify governance patterns that persist despite substantial contextual differences, suggesting their deep structural rather than contingent origins. The eleven focal countries were selected based on their potential to maximize variation across governance maturity, legislative tradition, regional affiliation, digital infrastructure level, and stage of economic development, while ensuring comprehensive regional coverage across the five continental clusters. Data for the comparative analysis were drawn from four primary sources: legislative and regulatory document analysis, examining constitutions, labour statutes, data protection laws, and AI policy documents in each focal country; analysis of professional and civil society governance instruments, including codes of practice, ethical guidelines, and civil society AI ethics statements; secondary literature synthesis, drawing on peer-reviewed scholarship, policy institute reports, and international organisation assessments; and expert practitioner consultation, drawing on published interviews, conference proceedings, and grey literature from HR professional associations across the continent. The comparative analysis examines each country across five governance dimensions: legislative and regulatory architecture; professional standards and occupational governance; digital infrastructure and technological readiness; cultural and philosophical governance traditions; and civil society and community governance mechanisms. This five-dimensional framework provides the analytical grid through which cross-national comparisons are structured and through which the AAHGT typology is derived.

## Results and Discussion

The International Finance Corporation ( IFC, 2022) estimates that AI adoption among Sub-Saharan African enterprises has grown at an annual rate exceeding 25% since 2018, driven by mobile-first digital infrastructure, a young and increasingly digitally literate workforce, and competitive pressures from both local and multinational technology providers. AI tools for recruitment screening, performance monitoring, workforce scheduling, and learning personalisation are now deployed by major employers across the banking, telecommunications, retail, extractive, and public administration sectors. Yet the governance frameworks that should ensure these tools operate equitably, transparently, and in conformity with workers’ rights remain nascent, fragmented, and in most countries, absent altogether.

### Southern Africa: Regulatory Leadership and Implementation Tensions

**South Africa**

South Africa occupies a distinctive position in the continental AI HRM governance landscape as the country with the most developed legislative and professional standards infrastructure. The Employment Equity Act 55 of 1998 (EEA), the Protection of Personal Information Act 4 of 2013 (POPIA), and the Labour Relations Act 66 of 1995 collectively constitute a regulatory framework that, while predating the current generation of AI workforce tools, imposes substantive obligations on their deployment. POPIA’s provisions on automated decision-making (Section 71), its requirement for lawful processing of personal information, and the Information Regulator’s emerging enforcement activity create a de facto AI governance regime that is more operationally developed than those found in most other African countries.

The South African Board for People Practices (SABPP) has issued guidance on ethical AI in HRM ( SABPP, 2022), and the Presidential Commission on the Fourth Industrial Revolution ( PC4IR, 2020) provided a policy framework linking AI governance to employment equity and skills development imperatives. Nevertheless, significant implementation gaps persist. The EEA’s provisions have not been explicitly extended to AI-specific employment assessment scenarios; the Information Regulator remains under-resourced relative to its mandate; and academic validation of commercially deployed AI workforce tools remains critically inadequate. South Africa’s governance posture is best characterised as legislatively advanced but implementationally constrained.

**Botswana**

Botswana presents a contrasting case within the Southern African Development Community (SADC) cluster. Botswana’s economy, anchored historically in diamond mining and increasingly in financial services and tourism, has embraced a government-led digital transformation agenda through the Smart Botswana National ICT Masterplan 2015–2019 and the more recent Digital Transformation Roadmap. The Data Protection Act of 2018, modelled closely on GDPR principles, provides data governance infrastructure that implicitly constrains AI workforce data processing. However, there is no equivalent to South Africa’s EEA that specifically addresses equity in employment decisions, reflecting Botswana’s different post-independence political settlement, which is one less shaped by apartheid-era labour market exclusion and more by a developmental state philosophy that prioritises growth with employment equity achieved through educational and skills investment rather than legislative mandates.

The Botswana HR professional community, organised through the Botswana Institute of Human Resource Management (BIHRM), has begun engaging with AI governance questions, but formal professional standards for AI HRM practices remain absent. The country’s relatively small formal economy and high labour informality rates mean that the governance gap between formal regulatory frameworks and the employment contexts in which AI tools are deployed is less acute than in South Africa, but the absence of proactive governance infrastructure means Botswana is poorly positioned for the rapid AI adoption that its digital transformation ambitions imply.

### East Africa: Innovation-Led Governance and Communal Ethics

**Kenya**

Kenya has emerged as one of Africa’s most dynamic AI governance innovators, driven by its position as a regional technology hub that is anchored by Nairobi’s “Silicon Savannah” ecosystem and by a regulatory culture that has sought to position Kenya as a centre for responsible technology development on the continent. The Kenya Data Protection Act 2019, informed by GDPR principles but adapted to Kenyan institutional realities, creates a data governance foundation with direct implications for AI HRM. The Office of the Data Protection Commissioner (ODPC), established under the Act, has issued sector-specific guidance addressing automated profiling and the rights of data subjects subject to algorithmic employment decisions. Kenya’s National AI Strategy (2021) represents one of the few dedicated AI policy frameworks on the continent that explicitly addresses employment applications, calling for algorithmic impact assessments in HR contexts, transparency obligations for automated decision systems, and the development of AI literacy standards for HR professionals. The Institute of Human Resource Management Kenya (IHRM Kenya) has begun developing professional guidance on AI in HRM, drawing on international frameworks while seeking to integrate the Harambee philosophy of cooperative self-help as an ethical orientation for AI governance, whilst emphasising worker participation in AI system design and community benefit as governance criteria alongside individual rights protection.

**Rwanda**

Rwanda’s AI governance approach reflects the distinctive political economy of a developmental state that has achieved remarkable economic growth through centralised, technology-enabled public sector innovation. The Rwanda Data Protection and Privacy Law (2021) provide a comprehensive data governance framework, while Rwanda’s National AI Policy (2023) explicitly prioritises AI deployment in public sector service delivery and workforce management as a driver of the country’s Vision 2050 development goals. Rwanda’s governance approach is characterised by strong state capacity, rapid implementation, and a technocratic orientation that tends to prioritise efficiency gains from AI over precautionary worker protection measures. The Rwanda Workforce Development Authority (WDA) has incorporated AI upskilling objectives into national technical and vocational education frameworks, and the country’s high mobile money penetration and digital literacy levels provide an enabling environment for AI workforce tool adoption. However, the concentration of governance authority in state institutions, combined with limited civil society independence, raises questions about the degree to which Rwanda’s AI governance adequately safeguards workers’ rights as distinct from the state’s developmental productivity objectives. The governance framework is technically advanced but civil society accountability mechanisms are underdeveloped by comparative standards.

### West Africa: Pluralist Traditions and Emerging Regulatory Architecture

**Nigeria**

Nigeria, as Africa’s most populous country and largest economy by GDP, presents a governance case of continental significance. The National Information Technology Development Agency (NITDA) of Nigeria has been the primary governance actor in the AI space, issuing the Nigeria Data Protection Regulation (NDPR) 2019, which is subsequently strengthened by the Nigeria Data Protection Act 2023 and an AI Policy Roadmap (2022) that identifies employment applications as a priority governance domain. The National Information Technology Development Agency Act 2007 provides NITDA’s statutory mandate, though questions about institutional jurisdiction over AI employment decisions, which also fall under the Labour Act Cap L1 and the Employee Compensation Act 2010, have created regulatory fragmentation. Nigeria’s AI governance landscape is further shaped by the country’s complex federalism: labour regulation is a concurrent legislative matter under the 1999 Constitution, meaning that state-level variations in employment practice governance are constitutionally possible, though in practice federal standards tend to dominate. The Chartered Institute of Personnel Management of Nigeria (CIPM) has developed an AI Ethics Charter for HR Practitioners (2023) that represents one of the most substantive professional governance documents in West Africa, drawing on both international frameworks and indigenous Yoruba, Igbo, and Hausa ethical traditions as legitimating resources for AI ethics governance.

**Ghana**

Ghana’s approach to AI HRM governance reflects the country’s reputation as West Africa’s most stable democracy and its aspiration to become a technology and innovation hub for the sub-region. The Data Protection Act 2012 (Act 843), administered by the Data Protection Commission (DPC), provides an established data governance infrastructure, though it predates the current generation of AI systems and requires updating to address algorithmic processing specifically. Ghana’s National AI Strategy, published in 2023, is notable for its explicit engagement with African values as governance foundations, including the concept of Anidaso (hope and forward-looking vision in Akan philosophy) as an orientation for technology development that serves both present and future generations. Ghana’s HR professional community, organised through the Institute of Human Resource Management Ghana (IHRM Ghana), has developed competency standards that include AI literacy as a dimension of contemporary HR practice. The Ghana Employers Association has published guidance on responsible AI adoption in workforce management, reflecting an employer-led governance initiative that complements the state regulatory framework. Ghana’s polycentric governance architecture, which is characterised by complementary contributions from state regulators, professional associations, employer bodies, and civil society, represents a model of pluralist governance that aligns well with the theoretical framework developed in Section 2.

**Senegal**

Senegal’s AI HRM governance approach reflects both its Francophone legal heritage, which includes French-inspired civil law structures and GDPR-influenced data protection through the Loi 2008–12 sur la Protection des Données Personnelles and its subsequent updates, as well as its active engagement with the African Union’s continental governance agenda. Senegal has been a particularly influential voice in the development of the African Union’s AI governance frameworks, contributing to the AU Data Policy Framework (2022) and positioning Dakar as a hub for pan-African AI ethics discourse. The country’s Commission de Protection des Données Personnelles (CDP) has begun developing AI-specific guidance, including preliminary thinking on automated employment decision systems. Senegal’s governance distinctiveness lies partly in its philosophical tradition. The Wolof concept of Teranga, which is a philosophy of generous hospitality, social responsibility, and mutual care, has been invoked by Senegalese governance scholars and civil society actors as an indigenous ethical framework for AI development that centres communal benefit over individual commercial gain. This philosophical resource, combined with Senegal’s strong civil society sector and Francophone academic networks, creates a governance environment characterised by sophisticated ethical deliberation even in the absence of comprehensive AI-specific legislation.

### North Africa: Hybrid Governance and Regional Connectivity

**Egypt**

Egypt’s AI HRM governance approach reflects the country’s unique position at the intersection of African, Arab, and Mediterranean governance traditions, combined with a strong state orientation toward technology-led economic modernisation. Egypt’s National AI Strategy (2021) is one of the most comprehensive on the continent, articulating a governance vision that balances AI-enabled productivity enhancement with social protection considerations. The Personal Data Protection Law (PDPL 151/2020), which draws on GDPR architecture, provides data governance infrastructure for AI workforce tool deployment, and Egypt’s Ministry of Communications and Information Technology (MCIT) has developed AI governance guidelines for the private sector that include workforce management applications. Egypt’s large formal labour market, which is anchored by public sector employment, manufacturing, and services, includes its substantial HR professional community, known as the Egyptian Society for Human Resources Management (ESHRM), create conditions for meaningful professional governance engagement with AI in HRM. ESHRM has developed an AI Ethics Framework for HR Practitioners (2022) that draws explicitly on the ancient Egyptian concept of Ma’at (the principle of truth, justice, and cosmic order) as a philosophical foundation for AI governance, positioning this as a distinctively Egyptian contribution to the global AI ethics discourse. This philosophical framing resonates with Gwagwa et al.’s (2022) argument for the development of governance frameworks grounded in African philosophical traditions rather than exclusively Western ethical concepts.

**Morocco**

Morocco presents a distinctive governance profile characterised by deep integration with European regulatory frameworks, which reflects the country’s Association Agreement with the European Union and its aspiration for eventual EU market integration, alongside active engagement with pan-African governance institutions. The Commission Nationale de Controle de la Protection des Donnees a Caractere Personnel (CNDP) administers Morocco’s Data Protection Law (09–08), and the country’s National Digital Strategy (2030) explicitly identifies AI governance as a priority domain. Morocco’s HR professional community, through the Association Marocaine des Directeurs des Ressources Humaines (AMDRH), has developed professional guidelines on algorithmic management that are among the most technically detailed in North Africa. Morocco’s governance trajectory is distinctive in its dual alignment: drawing on EU regulatory models for technical governance standards while simultaneously engaging with the African Union’s continental governance agenda and with Moroccan-specific philosophical traditions, including the Amazigh concept of Tafra (collective flourishing) as an indigenous ethical resource. This dual alignment creates a governance bridge between European and African regulatory traditions that positions Morocco as a potential interlocutor in continental governance harmonisation processes.

### Central Africa: Nascent Frameworks and Institutional Capacity Challenges

**Cameroon**

Cameroon’s AI HRM governance landscape reflects the country’s bicultural Francophone-Anglophone institutional heritage, its position as Central Africa’s largest economy outside the Democratic Republic of Congo, and the governance constraints imposed by a challenging political environment. The Law No. 2010/012 on Cybersecurity and Cybercriminality provides a rudimentary digital governance framework, though it predates AI-specific governance challenges and lacks provisions relevant to algorithmic employment decisions. Cameroon’s National Digital Plan (NDP) 2020–2030 identifies AI as a priority technology domain but does not yet articulate an AI-specific governance framework.

The professional HR governance landscape in Cameroon, organised primarily through the Association des Professionnels en Ressources Humaines du Cameroun (APRHCAM), remains nascent in its engagement with AI governance. International development partners, including the International Labour Organization (ILO) and the World Bank’s Digital Development Partnership, have been important external actors in initiating AI governance dialogue in Cameroon, a pattern consistent with the broader continental dynamic of international development finance institutions shaping AI policy trajectories identified in Section 9. Cameroon’s governance posture is best characterised as nascent, with international partnership providing the primary governance stimulus in the absence of robust domestic regulatory capacity.

**Democratic Republic of Congo**

The Democratic Republic of Congo (DRC) presents perhaps the most acute governance challenge in the continental comparison. Despite possessing some of the world’s most significant deposits of cobalt and coltan, which are critical materials for AI hardware supply chains, the DRC’s AI HRM governance infrastructure is among the least developed on the continent. The country’s ongoing conflict dynamics, limited state institutional capacity outside Kinshasa, high labour informality, and low digital literacy collectively constrain the conditions under which formal AI governance frameworks could be effectively implemented even if enacted. The Loi-cadre n. 013/2022 sur les Technologies de l’Information et de la Communication provides a promising digital governance foundation, but HRM-specific provisions are absent.

The DRC case illustrates a fundamental governance paradox: countries that are most directly implicated in AI supply chains through mineral extraction, and whose workers are most exposed to AI-enabled surveillance and performance monitoring in extractive industry contexts, often have the least governance capacity to protect those workers from algorithmic harm. This paradox has important implications for how continental governance harmonisation is designed, which suggests that capacity building, resource transfer, and differentiated implementation timelines are prerequisites for meaningful governance inclusion of the continent’s most institutionally constrained countries.

## Continental Patterns and Axes of Divergence

### Three Continental Governance Patterns

The comparative analysis reveals as exemplified in Table 1 three patterns that characterise AI HRM governance across the African continent, transcending regional boundaries and persisting across significant institutional diversity. The first pattern is the primacy of data protection law as a de facto AI HRM governance instrument. Across all eleven focal countries, data protection legislation, whether GDPR-inspired (South Africa, Kenya, Rwanda, Ghana, Senegal, Morocco, Egypt) or more rudimentary digital governance law (Botswana, Nigeria, Cameroon, DRC), constitutes the primary existing legal instrument with direct applicability to AI workforce tool deployment. In the absence of dedicated AI employment regulation, data protection provisions governing automated decision-making, purpose limitation, data minimisation, and data subject rights function as the primary accountability mechanism for AI HRM systems. This pattern reflects the global trajectory of AI governance through data protection frameworks ( Cihon et al., 2020) but takes a distinctive form in Africa, given the relatively recent enactment of many data protection laws and the limited enforcement capacity of data protection authorities. The second pattern is the significant influence of international development finance institutions (DFIs) and multilateral organisations on national AI policy trajectories. The World Bank’s Digital Economy for Africa (DE4A) initiative, the African Development Bank’s Digital Infrastructure Programme, the IFC’s AI and Digital Finance programmes, and UNESCO’s AI ethics activities have all shaped national AI policy agendas in ways that introduce external governance norms into domestic governance discourses. While this external influence has accelerated AI governance development in capacity-constrained countries, it also risks producing governance frameworks that are externally legitimised but domestically thin, lacking the indigenous philosophical grounding and civil society engagement that would make them meaningful in practice. The third pattern is the tension between aspirational digital transformation agendas and institutional capacity constraints. Across the continent, AI governance is located within broader digital transformation narratives that frame AI adoption as a developmental imperative that maps a pathway to leapfrogging traditional development stages and achieving Vision-level national goals. These aspirational frameworks generate optimistic AI governance postures that prioritise enabling conditions over precautionary regulation, creating a systematic risk that governance development lags adoption rates. The eleven focal countries all exhibit some version of this tension, though its intensity varies with institutional capacity: South Africa, Kenya, and Rwanda exhibit the strongest institutional capacity to manage the tension; Cameroon and the DRC exhibit the weakest.

**Table 1.** Three Continental AI HRM Governance Patterns: Features and Country Distribution.

Continental Pattern	Key Mechanism	Focal Countries	Governance Risk
**Data protection law as a de facto AI HRM governance instrument**	GDPR-inspired data protection provisions (automated decision-making, purpose limitation, data subject rights) applied to AI workforce tools in the absence of dedicated employment AI regulation	All 11 focal countries: strongest in South Africa, Kenya, Rwanda, Ghana, Senegal, Morocco, Egypt	Limited enforcement capacity; provisions not explicitly calibrated to AI employment contexts; accountability gaps for algorithmic decisions
**International DFI and multilateral influence on national AI policy**	World Bank DE4A, AfDB Digital Infrastructure, IFC, and UNESCO AI ethics activities introducing external responsible AI norms into domestic governance discourses	Most pronounced in capacity-constrained countries: Cameroon, DRC, Botswana, Senegal, Rwanda	Externally legitimated but domestically thin frameworks; weak indigenous philosophical grounding; low civil society engagement
**Tension between aspirational digital transformation and institutional capacity constraints**	National AI and digital economy visions framing AI adoption as a developmental leapfrogging imperative, creating enabling-oriented governance postures that prioritise deployment over precaution	All 11 countries; tension most acute in Cameroon and DRC; best managed in South Africa, Kenya, and Rwanda	Governance systematically lags adoption; workers are exposed to unvalidated AI employment tools before protective frameworks are in place

*Note*: DFI = Development Finance Institution; DE4A = Digital Economy for Africa (World Bank); AfDB = African Development Bank; IFC International Finance Corporation; DRC = Democratic Republic of Congo.

**Table 2.** Three Axes of Divergence in African AI HRM Governance.

Axis of Divergence	High-Specificity/Active Pole	Low-Specificity/Nascent Pole	Governance Implication
**Axis 1: Regulatory specificity directed at AI in employment decisions**	South Africa (EEA + POPIA s.71), Kenya (ODPC automated profiling guidance), Nigeria (NDPR/NDP)	DRC, Cameroon, Botswana — no employment-specific AI provisions; general labour law only	Countries at the nascent pole lack operative accountability for algorithmic employment decisions; workers have no recourse mechanism when AI-driven assessments produce discriminatory outcomes
**Axis 2: Incorporation of African philosophical traditions into AI ethics frameworks**	South Africa (Ubuntu), Nigeria (Omoluabi), Ghana (Anidaso), Senegal (Teranga), Egypt (Ma’at)	Rwanda, Botswana, Cameroon, DRC — primarily international standards (GDPR, OECD, UNESCO) without indigenous philosophical grounding	Frameworks at the nascent pole risk perceived illegitimacy and low voluntary compliance; culturally embedded frameworks generate higher community resonance and are more likely to be meaningfully implemented
**Axis 3: Role of regional economic communities (RECs) in harmonising AI governance**	EAC (Kenya-Rwanda bilateral template); ECOWAS (supplementary data protection act)	CEMAC (no substantive AI governance engagement); Arab Maghreb Union (constrained by political tensions); SADC and COMESA (reliant on existing digital economy frameworks)	Uneven REC engagement creates a fragmented continental governance architecture; cross-border AI HRM deployments face inconsistent regulatory requirements that may incentivise regulatory arbitrage

*Note*: EEA = Employment Equity Act 55 of 1998 (South Africa); POPIA = Protection of Personal Information Act 4 of 2013 (South Africa); ODPC = Office of the Data Protection Commissioner (Kenya); NDPR = Nigeria Data Protection Regulation; NDP = National Data Protection (Nigeria); EAC = East African Community; ECOWAS = Economic Community of West African States; CEMAC = Economic and Monetary Community of Central Africa; SADC = Southern African Development Community; COMESA = Common Market for Eastern and Southern Africa.

### Three Axes of Divergence

Against these continental patterns, three principal axes of divergence structure the cross-national variation in AI HRM governance approaches, as illustrated in Table 2. The first axis concerns the degree of regulatory specificity directed at AI in employment decisions. At one pole, South Africa’s EEA and POPIA, Kenya’s ODPC guidance on automated profiling, and Nigeria’s NDPR/NDP framework create relatively specific governance obligations for AI employment applications. At the other pole, the DRC, Cameroon, and Botswana lack employment-specific AI governance provisions, relying on general labour law and emerging data protection frameworks. The intermediate position is occupied by Ghana, Rwanda, Senegal, Egypt, and Morocco, where AI governance frameworks address employment applications at the level of principles and strategic priorities but without the operational specificity required for effective regulatory accountability. The second axis concerns the extent to which African philosophical traditions are incorporated into AI ethics frameworks. Some countries, including Nigeria (Omoluabi), Ghana (Anidaso), Senegal (Teranga), Egypt (Ma’at), and South Africa (Ubuntu), have developed governance frameworks that explicitly invoke indigenous philosophical traditions as ethical foundations, creating governance documents that are not merely technical but culturally embedded. Other countries, including Rwanda, Botswana, Cameroon, and the DRC, have produced governance frameworks that draw primarily on international standards (GDPR, UNESCO AI principles, OECD AI Principles) without significant indigenous philosophical grounding. This divergence has implications for governance legitimacy and civil society uptake: frameworks grounded in familiar cultural values tend to generate higher community resonance and voluntary compliance than frameworks perceived as externally imposed technical standards.

The third axis concerns the role of regional economic communities (RECs) in harmonising AI governance across national borders. The East African Community (EAC) has been the most active in AI governance harmonisation, with Kenya and Rwanda’s bilateral digital economy agreements providing a template for broader EAC-level coordination. The Economic Community of West African States (ECOWAS) has developed a supplementary act on personal data protection that provides a partial governance harmonisation instrument. SADC and the Common Market for Eastern and Southern Africa (COMESA) have been slower to develop AI-specific governance instruments, relying primarily on their existing digital economy and data protection frameworks. The Arab Maghreb Union provides a potential harmonisation vehicle for North African countries, though its institutional activity has been constrained by member-state political tensions. In Central Africa, CEMAC (Economic and Monetary Community of Central Africa) has not yet engaged substantively with AI governance.

### The African AI HRM Governance Typology (AAHGT)

**Figure 1.**
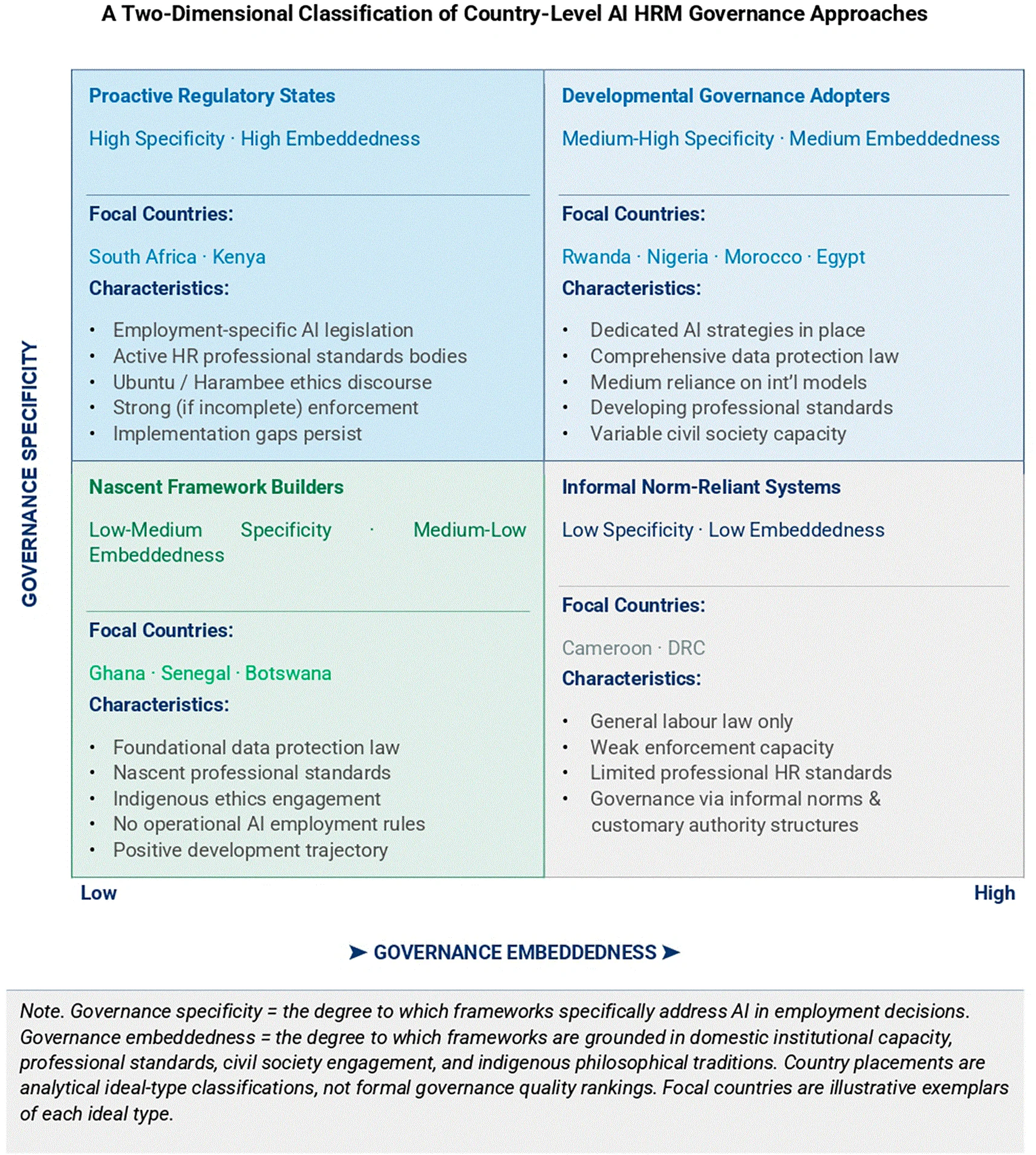
The African AI HRM Governance Typology (AAHGT): Four Ideal Types Positioned on Two Governance Dimensions.

Drawing on the comparative analysis presented in this article introduces the African AI HRM Governance Typology (AAHGT) as an original analytical contribution. The AAHGT classifies country-level governance approaches across four ideal types defined by two underlying dimensions: governance specificity (the degree to which governance frameworks specifically address AI in employment decisions) and governance embeddedness (the degree to which governance frameworks are grounded in domestic institutional capacity, professional standards, civil society engagement, and indigenous philosophical traditions). The four ideal types are presented in Figure 1 and described below.

Proactive Regulatory States, as stated in Table 3 are characterised by high governance specificity with legislation or regulatory guidance that directly addresses AI in employment decisions. These are combined with high governance embeddedness, reflected in domestic institutional capacity, active professional standards bodies, civil society engagement, and explicit invocation of indigenous philosophical traditions. South Africa and Kenya are the clearest exemplars. Both countries have enacted data protection legislation with specific automated decision-making provisions, professional HR standards bodies engaged with AI governance, and developing traditions of Ubuntu or Harambee-informed AI ethics discourse. Both also exhibit governance implementation gaps that prevent their formal frameworks from fully protecting workers from algorithmic harm, reinforcing the distinction between governance architecture and governance effectiveness.

**Table 3.** The African AI HRM Governance Typology (AAHGT): Four ideal types with focal country classifications.

Ideal Type	Governance Specificity	Governance Embeddedness	Focal Countries
Proactive Regulatory States	High	High	South Africa, Kenya
Developmental Governance Adopters	Medium-High	Medium	Rwanda, Nigeria, Morocco, Egypt
Nascent Framework Builders	Low-Medium	Medium-Low	Ghana, Senegal, Botswana
Informal Norm-Reliant Systems	Low	Low	Cameroon, DRC

### Developmental Governance Adopters

Developmental Governance Adopters have developed substantive AI governance frameworks often through dedicated AI strategies or comprehensive data protection legislation, but their governance is characterised by medium embeddedness: significant reliance on international governance models, developing professional standards infrastructure, and variable civil society capacity. Rwanda exemplifies the state-centric variant of this type: technically sophisticated governance, driven by a capable developmental state, but with limited civil society independence. Nigeria represents the pluralist variant: a complex multi-actor governance environment with significant professional and civil society engagement but fragmented by federal institutional architecture and jurisdictional overlap. Morocco and Egypt occupy the external-alignment variant, with governance frameworks shaped significantly by EU and international standards while developing indigenous philosophical grounding.

### Nascent Framework Builders

Nascent Framework Builders possess foundational governance infrastructure such as data protection legislation, nascent professional standards, and some civil society AI ethics engagement, but without governance frameworks that specifically address AI employment applications at an operational level. Ghana, Senegal, and Botswana are representative exemplars. These countries are actively constructing governance frameworks, often in dialogue with both international standards and indigenous philosophical traditions but have not yet achieved the governance specificity of Proactive Regulatory States or Developmental Governance Adopters. The trajectory of governance development in this category is positive, and several countries in this type, particularly Ghana and Senegal, exhibit governance innovation that may produce distinctive contributions to the continental governance landscape.

### Informal Norm-Reliant Systems

Informal Norm-Reliant Systems lack both governance specificity for AI employment applications and the domestic institutional embeddedness required for effective governance. Formal governance frameworks that exist such as general labour law, rudimentary digital governance statutes, are poorly enforced, and professional standards infrastructure and civil society AI governance capacity are weakly developed. Cameroon and the DRC exemplify this type, though with important differences: Cameroon has greater institutional capacity and is actively building governance frameworks with international partner support, while the DRC’s governance constraints are more acute and persistent. In Informal Norm-Reliant Systems, governance of AI in workforce management occurs primarily through informal norms such as employer self-regulation, community social expectations, and the implicit governance provided by customary authority structures, rather than through formal regulatory frameworks.

## Implications for Continental Governance Harmonisation

### The African Union’s Digital Transformation Strategy

The African Union’s Digital Transformation Strategy for Africa (2020–2030) identifies AI governance as a priority domain for continental coordination, calling for the development of a pan-African AI framework that harmonises national approaches while respecting subsidiarity and member state diversity. The AU’s Data Policy Framework (2022) provides a partial harmonisation instrument for data governance, but a comprehensive AI HRM governance framework that addresses employment applications, worker rights, algorithmic fairness, and the specific equity challenges of African labour markets remains to be developed.

The AAHGT typology provides the AU with a diagnostic instrument for sequencing harmonisation efforts according to country governance maturity. Harmonisation efforts directed at Proactive Regulatory States can focus on governance quality improvement aimed at strengthening implementation capacity, updating legislation for AI-specific scenarios, and deepening indigenous philosophical grounding in governance frameworks. Efforts directed at Developmental Governance Adopters can focus on governance specificity enhancement such as developing AI employment-specific regulatory guidance within existing data protection and labour law frameworks. Efforts directed at Nascent Framework Builders can focus on institutional capacity development inclusive of supporting professional standards bodies, civil society AI governance organisations, and regulatory authorities. Efforts directed at Informal Norm-Reliant Systems must prioritise foundational governance infrastructure development, with realistic recognition that comprehensive AI HRM governance is a medium-to-long term objective contingent on broader institutional strengthening.

### The African Continental Free Trade Area

The AfCFTA, which entered into force in 2021 and encompasses the world’s largest free trade area by number of participating countries, has significant implications for AI HRM governance harmonisation. As AfCFTA implementation advances, particularly the Protocol on Investment and the emerging Protocol on Digital Trade, the cross-border deployment of AI workforce tools by pan-African employers will become more prevalent. This cross-border deployment will expose governance gaps and inconsistencies across the AAHGT typology, creating both regulatory arbitrage risks and harmonisation incentives.

Regulatory arbitrage refers to where employers deploy AI workforce tools through subsidiaries incorporated in lower-governance jurisdictions to avoid more stringent obligations in higher-governance jurisdictions, is a particular risk given the governance maturity gradient between Proactive Regulatory States and Informal Norm-Reliant Systems. AfCFTA governance provisions that establish minimum AI HRM governance standards, including requirements for AI tool validation, automated decision-making disclosure, and worker rights to explanation and appeal, could mitigate this risk by creating a regulatory floor that applies across participating states. The ILO’s tripartite governance model provides a procedural architecture for developing these minimum standards through the kind of multi-stakeholder deliberation that polycentric governance theory identifies as essential for effective AI governance.

### Regional Economic Community Governance Coalitions

Given the AU’s broad mandate and the significant governance maturity diversity across its 54 member states, regional economic community (REC)-level governance coalitions offer a more tractable pathway for near-term harmonisation. The EAC’s digital economy integration agenda provides the most advanced template, with Kenya and Rwanda’s bilateral frameworks offering a scalable model for EAC-wide AI HRM governance standards. ECOWAS’s supplementary data protection act provides a foundation for West African harmonisation that could be extended to cover AI employment applications, drawing on Nigeria’s and Ghana’s professional governance innovations.

SADC presents a distinctive harmonisation opportunity given South Africa’s governance leadership and the potential for South African institutional models, such as the SABPP, POPIA’s Information Regulator, the EEA’s equity framework, to serve as governance templates for neighbouring states. The SADC Labour and Employment Ministers Committee provides an institutional venue for AI HRM governance harmonisation dialogue, and the SADC Protocol on Employment and Labour (2014) provides a partial normative foundation for extending harmonisation to AI-specific employment governance provisions.

For all REC-level harmonisation efforts, the AAHGT typology counsels against assuming that governance convergence toward South African or Kenyan models is either feasible or desirable for all member states in the short term. Differentiated implementation frameworks that allow Informal Norm-Reliant Systems to meet basic governance principles through accessible compliance pathways, while Proactive Regulatory States develop more stringent standards, are more likely to achieve genuine governance improvement across diverse institutional contexts than uniform standard-setting that is immediately compliant in name but unimplementable in practice.

## Conclusions

This article has presented the most comprehensive comparative analysis to date of AI HRM governance across the African continent, examining approaches in eleven focal countries across five regional clusters. The analysis has identified three continental patterns that transcend regional variation, namely, the primacy of data protection law, the influence of international DFIs, and the tension between aspirational digital transformation and institutional capacity. Furthermore, there are three principal axes of divergence concerning regulatory specificity, indigenous philosophical grounding, and regional economic community harmonisation activity. The African AI HRM Governance Typology (AAHGT) introduced in Section 10 provides an original analytical contribution that advances comparative AI governance scholarship by developing a classification framework grounded in African institutional realities rather than adapted from OECD-country comparisons. The typology’s four ideal types, namely, Proactive Regulatory States, Developmental Governance Adopters, Nascent Framework Builders, and Informal Norm-Reliant Systems, provide a governance maturity map that can guide continental harmonisation sequencing, international partner capacity building priorities, and national governance development planning. The article’s theoretical contributions are threefold. The integration of Comparative Institutionalism, Polycentric Governance Theory, and African Philosophy provides a richer analytical framework for understanding African AI governance than any single theory affords. Secondly, the identification of African philosophical traditions, such as Ubuntu, Ujamaa, Harambee, Ma’at, Teranga, Anidaso, as active governance epistemologies rather than passive cultural contexts advance the decolonialisation of AI governance scholarship and points toward governance frameworks that are genuinely legitimate within African communities rather than merely technically compliant with international standards. Thirdly, the polycentric governance analysis, foregrounding the contributions of professional associations, civil society organisations, traditional governance institutions, and regional economic communities alongside state regulatory bodies, challenges state-centric governance narratives that underestimate the governance resources available in African contexts. Three priority areas for future research follow from this analysis. First, empirical governance effectiveness studies are needed across the eleven focal countries, moving beyond the governance architecture analysis provided here to assess whether existing frameworks constrain algorithmic harm in practice. This question requires data on worker experiences of AI-mediated HR decisions, employer compliance behaviour, and regulatory enforcement activity. Second, the governance role of African multinational employers, including Pan-African banks, telecommunications groups, and retail chains, warrants dedicated analysis: these actors deploy AI workforce tools across multiple AAHGT types simultaneously and their governance choices have outsized influence on Table 4 AI HRM governance norms across the continent. Third, the relationship between AfCFTA implementation and AI HRM governance harmonisation requires longitudinal tracking as the protocol on digital trade develops, with particular attention to whether trade liberalisation generates governance convergence or intensifies regulatory arbitrage dynamics. Together, these research priorities would substantially advance the knowledge base needed for African AI HRM governance to fulfil its promise, whilst ensuring that artificial intelligence in African workplaces serves the values of dignity, equity, and continental solidarity rather than merely the imperatives of technological efficiency and commercial return.

**Table 4.** Continental AI HRM Governance Harmonisation: Mechanisms, Actors, and Priority Actions.

Harmonisation Vehicle	Key Actor(s)	Current Status	Priority Actions for AI HRM Governance
**African Union Digital Transformation Strategy (AU-DTS)**	African Union Commission; AU Smart Cities and Digital Economy Division	Continental digital transformation framework in place; AI ethics principles under development; no binding AI employment governance instruments	Develop an AU Model Law on AI in Employment; establish continental AI workforce tool validation standards incorporating indigenous philosophical traditions; fund capacity-building for Nascent Framework Builders and Informal Norm-Reliant Systems
**African Continental Free Trade Area (AfCFTA)**	AfCFTA Secretariat; State Parties; digital trade working groups	Phase II negotiations include digital trade; cross-border labour mobility protocols are underdeveloped; no AI-specific employment provisions	Integrate AI HRM governance baseline standards into Phase II digital trade chapter; require mutual recognition of AI employment tool certifications as a trade facilitation measure; ensure AAHGT-differentiated compliance pathways prevent harmonisation from entrenching governance inequality
**Regional Economic Community (REC) Governance Coalitions**	EAC, ECOWAS, SADC, COMESA, Arab Maghreb Union, CEMAC Secretariats; national HR professional bodies (SABPP, IHRM-Kenya, CIPM-Nigeria)	EAC most active; ECOWAS data protection supplementary act, partial instrument; SADC, COMESA, CEMAC, Arab Maghreb Union, limited AI-specific engagement	Formalise Proactive Regulatory State–led governance coalitions within each REC; develop REC-level AI HRM governance minimum standards anchored in indigenous ethical traditions; establish cross-border data sharing agreements and joint enforcement protocols to address regulatory arbitrage

*Note*: AU-DTS = African Union Digital Transformation Strategy 2020–2030; AfCFTA = African Continental Free Trade Area; EAC = East African Community; ECOWAS = Economic Community of West African States; SADC = Southern African Development Community; COMESA = Common Market for Eastern and Southern Africa; CEMAC = Economic and Monetary Community of Central Africa; SABPP = South African Board for People Practices; IHRM = Institute of Human Resource Management (Kenya); CIPM = Chartered Institute of Personnel Management (Nigeria); AAHGT = African AI HRM Governance Typology.

## Ethical Approval Statement

Ethical approval was not deemed necessary for this study, as it did not involve live human participants, animal experimentation, or the handling of sensitive data. The research was limited to a qualitative comparative analysis in a form of literature review, analysis of publicly available information, or secondary analysis of previously published data, which does not warrant formal review by an ethical oversight committee.

## AI Disclosure Statement

During the preparation of this work, the author(s) used [ https://goblin.tools/Formalizer] to improve readability and grammar. After using this tool, the author(s) reviewed and edited the content as needed and take(s) full responsibility for the final publication.

## Data Availability

The Data for this Article Consists of Bibliographic References, which are Included in the References Section.
