# Supplementary material for: A Comparative Analysis of AI HRM Governance Approaches Across African Countries: Continental Patterns and Divergences
Source: F1000Res. 2026 Aug 31;15:1051. Originally published 2026 Jul 1. [Version 2] doi: 10.12688/f1000research.184277.2 (PMC13562992; doi:10.12688/f1000research.184277.2)
Supplement: Peer review history

# Reviewer report: Reviewer response for version 2

**DOI:** 10.5256/f1000research.207291.r516439

**Published:** 2026-09-04

**Authors:** Ravi Kumar Bommisetti

**Affiliations:** 1 Akal University, Bathinda, Punjab, India

After evaluating the revised manuscript in light of the authors' responses to the review comments, I find it suitable for indexing. I have no hesitation in recommending its acceptance.

Is the work clearly and accurately presented and does it cite the current literature?

Yes

If applicable, is the statistical analysis and its interpretation appropriate?

Partly

Are all the source data underlying the results available to ensure full reproducibility?

Yes

Is the study design appropriate and is the work technically sound?

Yes

Are the conclusions drawn adequately supported by the results?

Yes

Are sufficient details of methods and analysis provided to allow replication by others?

Partly

Reviewer Expertise:

NA

I confirm that I have read this submission and believe that I have an appropriate level of expertise to confirm that it is of an acceptable scientific standard.

# Reviewer report: Reviewer response for version 1

**DOI:** 10.5256/f1000research.203410.r499600

**Published:** 2026-07-22

**Authors:** Seema Bhakuni, Sandeep Saxena

**Affiliations:** 1 Doon Institute of Management and Research, Rishikesh, Uttarakhand, India; 2 Management, FS University, Shikohabad, Uttar Pradesh, India

The article focuses on the governance of Artificial Intelligence on Human Resource Management in 54 African countries out of which 11 from five regional clusters are selected by the author/s. The author wants to ensure the humane factors along with focussing on the use of AI.

 The method chosen is qualitative and based primarily on secondary data. Five key dimensions are focused upon which are legislative architecture, professional standards, digital infrastructure, philosophical traditions, and mechanisms established by civil society. The author/s specifies threefold theoretical contributions through the study which is the integration of Comparative Institutionalism, Polycentric Governance Theory, and African

 Philosophy for understanding African AI governance which is a better approach citing that a single theory might not be able to deliver results as clearly . The author identifies the African philosophical traditions that are genuinely legitimate within African communities rather than merely technically compliant with international standards. Thirdly, the author concludes with a comparative analysis for AI framework citing the significance of polycentric governance analysis, foregrounding the contributions of professional associations, civil society organisations, traditional governance institutions, and regional economic communities along with state regulatory bodies. 

 The author has given references up to the year 2022. The latest references can be added.(refer 1 to 3)

Is the work clearly and accurately presented and does it cite the current literature?

Partly

If applicable, is the statistical analysis and its interpretation appropriate?

Not applicable

Are all the source data underlying the results available to ensure full reproducibility?

Yes

Is the study design appropriate and is the work technically sound?

Yes

Are the conclusions drawn adequately supported by the results?

Yes

Are sufficient details of methods and analysis provided to allow replication by others?

Yes

Reviewer Expertise:

Human Resource Management, Labour law, Organisational Behaviour, Business Communication, Human Resource Development

We confirm that we have read this submission and believe that we have an appropriate level of expertise to confirm that it is of an acceptable scientific standard.

## References

[1] : Adoption of artificial intelligence in the HRM function within the South African telecommunication sector: a quantitative analysis using structural equation modelling. *Frontiers in Artificial Intelligence* .2026;9: 10.3389/frai.2026.1807550 10.3389/frai.2026.1807550 PMC1318384142164280

[2] : The impact of artificial intelligence on human resource management practices: An investigation. *SA Journal of Human Resource Management* .2025;23: 10.4102/sajhrm.v23i0.2960 10.4102/sajhrm.v23i0.2960

[3] : Integration of Artificial Intelligence into Human Resource Management in Manufacturing Enterprises: A Systematic Literature Review of Challenges, Approaches, and Evolution (2000–2025). *Sustainability* .2026;18(5) : 10.3390/su18052618 10.3390/su18052618

# Reviewer report: Reviewer response for version 1

**DOI:** 10.5256/f1000research.203410.r499598

**Published:** 2026-07-22

**Authors:** Ravi Kumar Bommisetti

**Affiliations:** 1 Akal University, Bathinda, Punjab, India

**STRENGTHS**

 1.Research Gap & Contribution: Excellent positioning. Claiming Africa as a site of "original governance innovation" rather than a "recipient" is novel and important. The 4 stated contributions are clear and significant.

 2.Theoretical Triangulation: The combination of Comparative Institutionalism + Polycentric Governance + African Philosophy is original. Ubuntu, Ujamaa, Harambee, Ma’at, Sankofa as "governance epistemologies" is the paper’s most distinctive contribution.

 3.Empirical Scope: 11 countries across 5 regional clusters is ambitious and credible. Use of MSSD + MDSD + QCA shows methodological rigor.Policy Relevance: Direct linkage to AU, AfCFTA, Digital Transformation Strategy gives the paper immediate policy impact.

**MAJOR Weaknesses & Critical Comments**

 1.The treatment of Comparative Institutionalism is under-developed. To address this, the authors should propose a framework for "institutional hybridity" and include one illustrative case showing the interaction of customary, colonial, and statutory law in AI HRM decisions. Citing Osei-Tutu (2022) or Nwauche (2015) on African legal pluralism would also strengthen this section.

 2.Grouping Ubuntu, Ujamaa, Harambee, Ma’at, and Sankofa under "African Philosophy" risks essentialism given their distinct regional and historical origins, so the authors should reframe them as "selected indigenous governance epistemologies across regions," justify their selection, and clarify whether concepts like Ubuntu are invoked by states or primarily by NGOs.

 3.The authors should clarify that they use a "qualitative comparative approach informed by QCA logic" rather than claiming QCA, provide a table justifying the selection of the 11 countries to address potential Anglophone bias, and acknowledge that reliance on secondary sources and published interviews limits insights into governance "in practice."

 4. Sections 4–8 currently read as descriptive country profiles rather than comparative analysis, so the authors should restructure them using the 5 governance dimensions as sub-headings and conclude each case with explicit cross-country comparisons, while also grounding claims about AI adoption with specific citations and firm-level examples, and elevating the "law on books vs law in action" implementation gap noted for South Africa into a cross-cutting analytical theme central to African governance.

 5.Trim the 700+ word introduction by 30% and relocate IFC stats and sector examples to Results. Update citations to include 2023–2024 works and define "erosions of worker dignity" using concrete dimensions like surveillance and algorithmic management.

 6. Elevate the AAHGT as the central contribution with a full typology matrix and a color-coded African map, illustrated with one country example per cell. The authors should also add "platform power" as a governance dimension and include a Limitations section addressing Anglophone bias, secondary data reliance, and policy volatility.

**Decision: Major Revision**

 Complete all tables and figures, restructure Results around the 5 dimensions, and clarify the method by dropping or properly executing QCA. The manuscript also requires a Limitations section and a more critical assessment of the role of DFIs in harmonizing versus fragmenting governance.

 I recommend major revisions. If the authors address the issues above, the manuscript could be suitable for indexing, subject to the Editor-in-Chief’s final decision.

Is the work clearly and accurately presented and does it cite the current literature?

Yes

If applicable, is the statistical analysis and its interpretation appropriate?

Partly

Are all the source data underlying the results available to ensure full reproducibility?

Yes

Is the study design appropriate and is the work technically sound?

Yes

Are the conclusions drawn adequately supported by the results?

Yes

Are sufficient details of methods and analysis provided to allow replication by others?

Partly

Reviewer Expertise:

Qualitative and Quantitative Research

I confirm that I have read this submission and believe that I have an appropriate level of expertise to confirm that it is of an acceptable scientific standard, however I have significant reservations, as outlined above.

# Response

**Published:** 2026-07-25

**Authors:** Samuel Bangura

**Affiliations:** Human Resource Management, Mangosuthu University of Technology, Umlazi, KwaZulu-Natal, South Africa

1. The treatment of Comparative Institutionalism is underdeveloped. To address this, the authors should propose a framework for "institutional hybridity" and include an illustrative case that shows the interaction among customary, colonial, and statutory law in AI HRM decisions. Citing Osei-Tutu (2022) or Nwauche (2015) on African legal pluralism would also strengthen this section. *THIS SECTION HAS BEEN UPDATED in the* *Theoretical Framework with the heading* *Institutional Hybridity Framework*

 2. Grouping Ubuntu, Ujamaa, Harambee, Ma’at, and Sankofa under "African Philosophy" risks essentialism given their distinct regional and historical origins, so the authors should reframe them as "selected indigenous governance epistemologies across regions," justify their selection, and clarify whether concepts like Ubuntu are invoked by states or primarily by NGOs. *THIS SECTION HAS BEEN UPDATED under the heading* *African Philosophy as Governance Epistemology. The concepts represent selected indigenous governance epistemologies across various regions. They are regarded as philosophical traditions that transcend superficial cultural references, constituting authentic governance epistemologies that shape the legitimisation, deliberation, and contestation of artificial intelligence regulation within diverse African contexts. Consequently, they are deemed to hold significant importance. It is also noteworthy that these concepts are invoked by state actors rather than solely by non-governmental organisations.*

 3. The authors should clarify that they use a "qualitative comparative approach informed by QCA logic" rather than claiming QCA, provide a table justifying the selection of the 11 countries to address potential Anglophone bias, and acknowledge that reliance on secondary sources and published interviews limits insights into governance "in practice."

*RESPONSE:* *While we recognised the recommendation of the reviewer, it is important to note that the 11 countries chosen to illuminate the main facet of the study; they were not merely chosen but purposively to highlight insights on the study. In this regard, only the QCA logic was updated*

 4. Sections 4–8 currently read as descriptive country profiles rather than comparative analysis, so the authors should restructure them using the 5 governance dimensions as sub-headings and conclude each case with explicit cross-country comparisons, while also grounding claims about AI adoption with specific citations and firm-level examples, and elevating the "law on books vs law in action" implementation gap noted for South Africa into a cross-cutting analytical theme central to African governance.

*RESPONSE:* *The comments here are highly recognised. However, while it seems that  the write-up is more descriptive country profiles rather than comparative analysis, the aim was to illuminate insight on the study based on country-to-country profiles*

 5. Trim the 700+ word introduction by 30% and relocate IFC stats and sector examples to Results. *This has been done as recommended.* Update citations to include 2023–2024 works and define "erosion of worker dignity" using concrete dimensions like surveillance and algorithmic management.

 RESPONSE: again while the suggestion is appreciated.

*RESPONSE: We write to affirm that the citations were researched and documented before application in the study, thus one can affirm that the cited works were well informed to address the discussion and therefore do not warrant further updates, which can be reserved for further studies within this domain*

 6. Elevate the AAHGT as the central contribution with a full typology matrix and a colour-coded African map, illustrated with one country example per cell.

*RESPONSE: The sub-Saharan facet of the study makes up for recommendations.*

 7.The authors should also add "platform power" as a governance dimension and include a Limitations section addressing Anglophone bias, secondary data reliance, and policy volatility.

*RESPONSE: The reality of the study is premised on the fact the anglophone is in the majority, and no purposeful bias is or was intended, and the nature of the study did allow for secondary data reliance, as the study did not aim for empirical or human involvement as participants.*
